# Angiotensin II-induced podocyte apoptosis is mediated by endoplasmic reticulum stress/PKC-δ/p38 MAPK pathway activation and trough increased Na^+^/H^+^ exchanger isoform 1 activity

**DOI:** 10.1186/s12882-018-0968-4

**Published:** 2018-07-13

**Authors:** Vanessa Gerolde Cardoso, Guilherme Lopes Gonçalves, Juliana Martins Costa-Pessoa, Karina Thieme, Bruna Bezerra Lins, Fernando Augusto Malavazzi Casare, Mariana Charleaux de Ponte, Niels Olsen Saraiva Camara, Maria Oliveira-Souza

**Affiliations:** 1 Laboratory of Renal Physiology, Department of Physiology and Biophysics, Institute of Biomedical Sciences, University of Sao Paulo, Sao Paulo, SP 05508-900 Brazil; 2 Laboratory for Transplantation Immunobiology, Department of Immunology, Institute of Biomedical Sciences, University of Sao Paulo, Sao Paulo, Brazil; 3 Laboratory of Carbohydrates and Radioimmunoassays (LIM-18), Medical School, University of Sao Paulo, Sao Paulo, Brazil

**Keywords:** Podocytes, Apoptosis, Angiotensin II, PKC-delta, p38 MAPK, NHE1

## Abstract

**Background:**

Angiotensin II (Ang II) contributes to the progression of renal diseases associated with proteinuria and glomerulosclerosis mainly by inducing podocyte apoptosis. In the present study, we investigated whether the chronic effects of Ang II via AT1 receptor (AT1R) would result in endoplasmic reticulum (ER) stress/PKC-delta/p38 MAPK stimulation, and consequently podocyte apoptosis.

**Methods:**

Wistar rats were treated with Ang II (200 ng·kg^−1^·min^−1^, 42 days) and or losartan (10 mg·kg^−1^·day^−1^, 14 days). Immortalized mouse podocyte were treated with 1 μM Ang II and/or losartan (1 μM) or SB203580 (0.1 μM) (AT1 receptor antagonist and p38 MAPK inhibitor) for 24 h. Kidney sections and cultured podocytes were used to evaluate protein expression by immunofluorescence and immunoblotting. Apoptosis was evaluated by flow cytometry and intracellular pH (pHi) was analyzed using microscopy combined with the fluorescent probe BCECF/AM.

**Results:**

Compared with controls, Ang II via AT1R increased chaperone GRP 78/Bip protein expression in rat glomeruli (*p* < 0.001) as well as in podocyte culture (*p* < 0.01); increased phosphorylated eIf2-α (*p* < 0.05), PKC-delta (*p* < 0.01) and p38 MAPK (*p* < 0.001) protein expression. Furthermore, Ang II induced p38 MAPK-mediated late apoptosis and increased the Bax/Bcl-2 ratio (*p* < 0.001). Simultaneously, Ang II via AT1R induced p38 MAPK-NHE1-mediated increase of pHi recovery rate after acid loading.

**Conclusion:**

Together, our results indicate that Ang II-induced podocyte apoptosis is associated with AT1R/ER stress/PKC-delta/p38 MAPK axis and enhanced NHE1-mediated pHi recovery rate.

## Background

Podocytes are highly specialized cells characterized by complex actin-rich foot processes that reside on the outside of the glomerular basement membrane (GBM). The foot processes interdigitate with the counterparts of neighboring cells to form a slit diaphragm composed of proteins, such as nephrin and p-cadherin. Under physiological conditions, podocytes play a critical role in the maintenance of the structure and function of the glomerular filtration barrier [1]. However, numerous studies have demonstrated a relevant contribution of podocytes in the pathogenesis and progression of chronic kidney diseases [2–7]. Along this line, slit diaphragm disruption and foot process effacement have been considered early manifestations of progressive podocyte damage and cell loss [8], resulting in glomerular hemodynamic disorders, proteinuria and glomerulosclerosis [9–12].

Angiotensin II (Ang II) is the major component of the renin-angiotensin system (RAS) and has numerous physiological functions. However, at high circulating concentrations, Ang II stimulates intrarenal RAS and induces glomerular injury, which progresses toward end-stage renal disease [7, 13]. Podocytes express both types of Ang II receptors (AT1R and AT2R) and have been shown to be target cells of the peptide [14]. Additionally, in vitro and in vivo studies have demonstrated that under high Ang II concentrations, podocytes showed decreased nephrin expression and increased number of apoptotic events [6, 15]. Although these mechanisms have not been thoroughly explored, previous studies have indicated that the effects of Ang II via AT1R activation on podocyte injury are related to reactive oxygen species (ROS) overproduction mediated by nicotinamide adenine dinucleotide phosphate (NADPH) oxidase [16, 17]. In other cell types, ROS production is closely related to ER stress, protein kinase C delta (PKC-δ) and p38 mitogen-activated protein kinase (p38 MAPK) activation, and together, these events are associated with apoptotic responses [18–21].

In turn, p38 MAPK also regulates mechanisms involved in intracellular pH (pHi) control [22], particularly by phosphorylating the Na^+^/H^+^ exchanger isoform 1 (NHE1) at serine sites (Ser 726 and Ser 729) [23]. NHE1 protein is ubiquitously distributed in the plasma membrane and in polarized epithelial cells; its mature form is localized almost exclusively in the basolateral membrane [24]. NHE1 regulates cellular pH and volume and participates in multiple cellular functions, including proliferation, migration and apoptosis [25]. We previously demonstrated that NHE1 is activated by Ang II in renal tubular cells [26, 27]. Grenier et al. [23] demonstrated potential link between NHE1 activity and alkalinization-mediated apoptosis in NHE1-transfected cells. Additionally, podocytes have been shown to express NHE1 [28] and undergo apoptosis when exposed to high concentrations of Ang II [6]. However, the mechanisms associated with Ang II/AT1R/NHE1 and podocyte apoptosis have not been elucidated.

In light of these evidences, we aimed to test the hypothesis that Ang II-induced podocyte apoptosis is associated with ER stress/PKC-δ/p38 MAPK activation and pHi changes. These findings will provide relevant information regarding the molecular mechanisms through which Ang II contributes to the progression of chronic kidney disease associated with podocyte injury.

## Methods

### Animal study design

Study was performed with the approval of the ETHICS COMMITTEE ON ANIMAL USE, Institute of Biomedical Sciences, University of Sao Paulo (CEUA-ICB/USP), Sao Paulo, Brazil (Protocol n^o^ 139/110/2011). Wistar rats (Animal Laboratory of the Department of Physiology and Biophysics, Institute of Biomedical Sciences, University of Sao Paulo, Sao Paulo, Brazil), weighing 160–200 g were housed in cages and maintained in a temperature-controlled room with a light-dark cycle of 12:12-h, with free access to tap water and standard rat chow for 2 weeks. To address the role of chronic Ang II on podocyte apoptosis in vivo, animal models have been described previously [7]. Briefly, rats (*n* = 6/group) were randomly assigned to sham surgery (control) or osmotic minipump insertion (Alzet model 2006, Alza, Mountain View, Calif., USA) for Ang II infusion (Tocris Bioscience, Bristol, UK) at 200 ng · kg-1 · min-1 for 42 days. An additional group was treated with losartan (AT1 receptor antagonist, 10 mg·kg^− 1^·day^− 1^, s.c.; DuPont 753, Merck Pharmaceuticals, Deepwater, NJ) or Ang II plus losartan for 14 days. For all surgical procedures, the animals were anesthetized with zoletil (50 mg/kg zolazepam and 50 mg/kg tiletamine) and virbaxyl (5 mg/kg xylazine; Virbac). After the treatment, the animals were again anesthetized, the kidneys were perfused [7] and euthanasia was performed by exsanguination. One kidney was fixed in 4% paraformaldehyde solution for immunofluorescence analysis.

### Cell culture

Study was performed with the approval of the ETHICS COMMITTEE ON ANIMAL USE, Institute of Biomedical Sciences, University of Sao Paulo (CEUA-ICB/USP), Sao Paulo, Brazil (Protocol n^o^ 673/14). A conditionally immortalized mouse podocyte cell line was developed by Prof. Dr. Karlhans Endlich, University of Heidelberg, Germany and generously donated by Prof. Dr. Niels Olsen Saraiva Camara, Institute of Biomedical Sciences, University of Sao Paulo. The cells were cultured as previously described [29, 30] and summarized here. To induce proliferation, podocytes were grown in 75-cm^2^ flasks coated with type I collagen and maintained in RPMI medium (Thermo Fisher Scientific INC, St Peters, MO, USA) supplemented with 30 U/ml mouse recombinant γ-interferon (Cell Sciences, Newburyport, MA, USA) at the permissive temperature (33 °C). To induce differentiation, podocytes were maintained at 37 °C without γ-interferon for 10–13 days. Finally, 2.0 × 10^4^ cells/well at passages 8–12 were used in experiments.

### Cell culture treatment and experimental design

Differentiated podocytes were treated with 1 μM Ang II (Tocris Bioscience) for 24 h. To examine the beneficial effects of losartan on Ang II-induced podocyte injury, differentiated cells were incubated with culture media containing 1 μM losartan (DuPont 753, Merck Pharmaceuticals) for 30 min, followed by incubation with Ang II plus losartan for 24 h. To inhibit NHE1 and p38 MAPK activity, cariporide (10 μM, Santa Cruz Biotechnology, Dallas, TX, USA) and SB203580 (0.1 μM, Merck Millipore, Temecula, CA, USA) were added for 30 min, followed by incubation with Ang II plus specific inhibitors for 24 h.

### Immunoblotting

Total proteins extracts from control and Ang-II and/or inhibitors treated podocytes were obtained using ice-cold RIPA buffer (BioRad, Sao Paulo, Brazil) with protease and phosphatase inhibitors (Sigma Aldrich St. Louis, MO, USA). Immunoblot analysis was performed on aliquots containing 30 μg/lane of proteins resolved in 10% SDS-PAGE as previously described [22, 27]. The following primary antibodies were used in this study: rabbit anti-AT1 receptor (1:1000, Merck Millipore); rabbit anti-chaperone GRP 78 (1:8000, StressMarq, Victoria, BC, CA); rabbit anti-phospho-eIf2-α (eukaryotic initiation factor 2, an ER stress marker, 1:1000, Cell Signaling, Denver, MA, USA); rabbit anti-phospho-PKC-δ (1:3000, Abcam, Cambridge, MA, USA); rabbit anti-p38MAPK and anti-phospho-p38MAPK (1:1000, Cell Signaling); rabbit anti-Bcl-2 (1:1000, Cell Signaling); rabbit anti-Bax (1:4000, Santa Cruz); mouse anti-NHE1 (1:2000, Abcam); rabbit anti-podocin (1:1000, Sigma Aldrich); rabbit anti-GAPDH (1:2000, Cell Signaling); mouse anti-β-actin (1:5000, Abcam); and horseradish peroxidase-conjugated goat secondary antibodies against rabbit and mouse (Jackson ImmunoResearch Laboratories, Baltimore, MD, USA). Protein expressions are shown as the ratio to the endogenous control, namely GAPDH or β- actin.

### Immunofluorescence microscopy

Control or treated podocytes cultured on glass coverslips were fixed with 2% paraformaldehyde in PBS (0.15 M NaCl containing 10 mM sodium phosphate buffer, pH 7.4) for 4 min and permeabilized with 0.1% Triton X-100 in PBS for additional 5 min. Then, the cells were blocked with 1% bovine serum albumin (BSA) and 0.2% neutral gelatin in PBS for 30 min at room temperature before overnight incubation at 4 °C with the specific primary antibody rabbit anti-podocin (1:100, Sigma Aldrich). In the next day, cells were washed three times with PBS and incubated with the secondary antibody goat anti-rabbit Alexa Fluor 568 (1:200, Jackson ImmunoResearch Laboratories) for 1 h at room temperature in the dark. Cells nuclei were stained with 4′6-diamidine-2′-phenylindole dihydrochloride (DAPI; Sigma Aldrich) for 5 min at room temperature.

Kidney sections with 3–4 μm were deparaffinized and blocked with 3% BSA in PBS for 1 h at room temperature, before overnight incubation at 4 °C with the specific primary antibodies mouse anti-nephrin (1: 200, R & D Systems, Minneapoles, MS, USA) and rabbit anti-GRP 78 (1:100, StressMarq). In the next day, sections were washed three times with PBS and incubated with the secondary antibody F (ab’) donkey anti-goat Alexa Fluor 488 (1:200, Thermo Fischer Scientific) and goat anti-rabbit Alexa Fluor 594 (1:200, Thermo Fischer Scientific) for 1 h at room temperature in the dark. Slides were mounted with Fluoroshield (Sigma Aldrich) and analyzed using a Zeiss LSM 788 confocal microscope equipped with a 63× objective Plan-Apochromat, zoom factor 1, a laser excitation of 546 nm to Alexa fluor 568 and 594 nm acquisition and 405 nm to DAPI. Analyses were performed by investigators blinded to the study groups.

### Annexin V and propidium iodide (PI) staining assay

Control and Ang II and/or SB203580 treated podocytes were trypsinized and 3 ml of BioLegend Cell Staining Buffer (BioLegend, San Diego, CA, USA) were added to the cell samples. The cell suspensions were centrifuged at 2500 rpm for 5 min, twice. Cells were resuspended in Annexin V Binding Buffer (BioLegend) and 1 × 10^5^ cells/ml were transferred to a cytometry sample tube containing 1 μL of FITC-Annexin V (100 μg/ml) and 0.3 μL of PI (5 μg/ml). Samples were incubated in the dark for 10 min and fluorescence was subsequently analyzed using a BD FACS Canto II Flow Cytometer (San Jose, CA, USA), calibrated to detect 10.000 events. Cells positive for Annexin V-FITC were considered apoptotic.

### Fluorescence pHi measurement

As described previously [22, 27, 31, 32], the functional activity of the Na^+^/H^+^ isoform 1 (NHE1) was measured fluorometrically in control or treated podocytes cultured on glass coverslips, by using a pH-sensitive fluorescent probe [2′, 7′-bis-(2-carboxyethyl)-5-(and-6)-carboxyfluorescein, acetoxymethyl ester (BCECF-AM); Molecular Probes, Eugene, OR, USA] combined with the high K^+^-nigericin method.

### Statistical analysis

Data are reported as the mean ± SEM. Statistical significance was determined by 1-way ANOVA with a Bonferroni post-hoc test for comparisons of multiple groups. The differences with *p* < 0.05 were considered statistically significant.

## Results

### Chronic effect of Ang II/AT1R on glomerular GRP 78 expression in rats

**Fig. 1:**
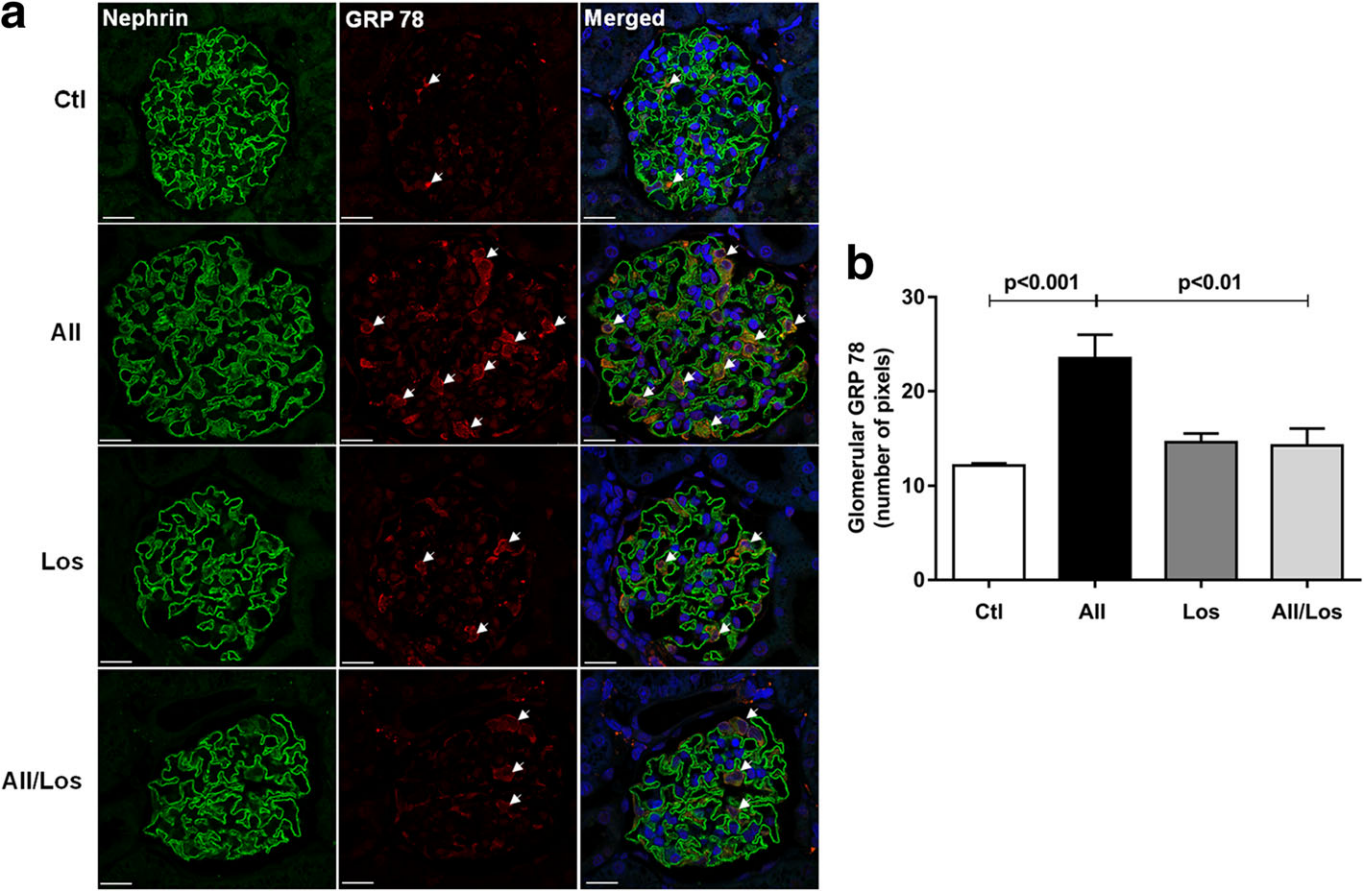
Ang II induces glomerular GRP 78 expression. **a** Immunofluorescence to detect the podocyte marker nephrin and GRP 78 in glomeruli from 6 weeks Ang II-treated rats. Original magnification, × 630; bar, 10 μm. **b** Quantification of glomerular GRP 78. Values represent the mean ± SEM (*n* = 6/group)

As shown in Fig. 1a, b, using rat kidney sections and immunofluorescence staining, we observed that treatment with Ang II for 6 weeks (42 days) induced an increase in glomerular GRP 78 expression. Although losartan treatment alone did not change glomerular GRP 78 expression, the AT1R antagonist reverted the Ang II effects [(fluorescence intensity) Ctl, 12.17 ± 0.21; AII, 23.5 ± 2.5; Los, 14.5 ± 0.93 and AII/Los, 14.23 ± 1.79 (*n* = 6)]**.** Nephrin staining was used to identify podocytes.

### Chronic treatment with Ang II leads to ER stress and PKC-δ phosphorylation in podocytes

**Fig. 2:**
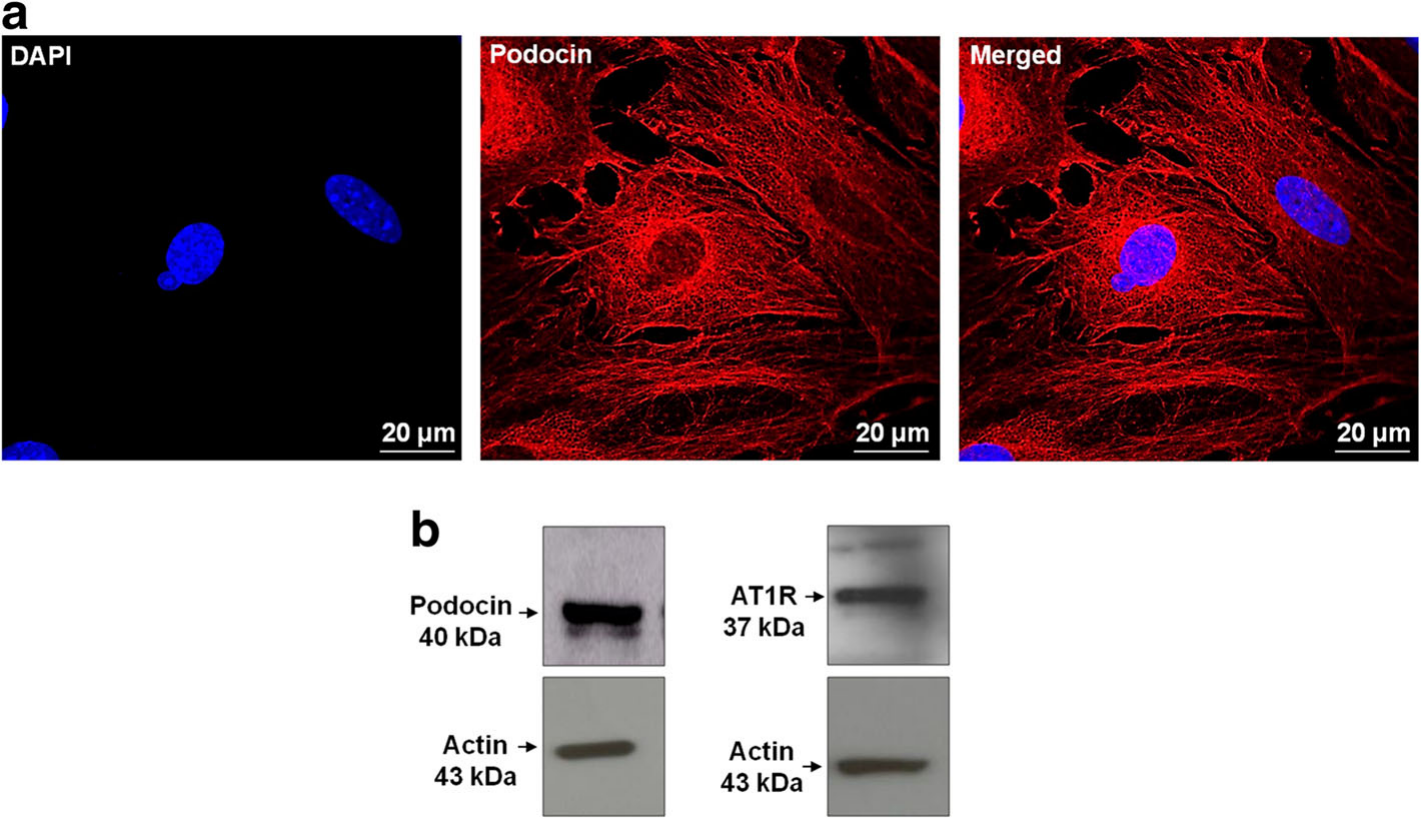
**a** Immunofluorescence staining of podocin in conditionally immortalized mouse podocytes. Podocin is labeled in red and DAPI (nuclei) in blue. Original magnification, × 630; bar, 20 μm; *n* = 3. **b** Representative bands of podocin and angiotensin II receptor 1 (AT1) proteins expression. Actin was used as internal control, *n* = 3

**Fig. 3:**
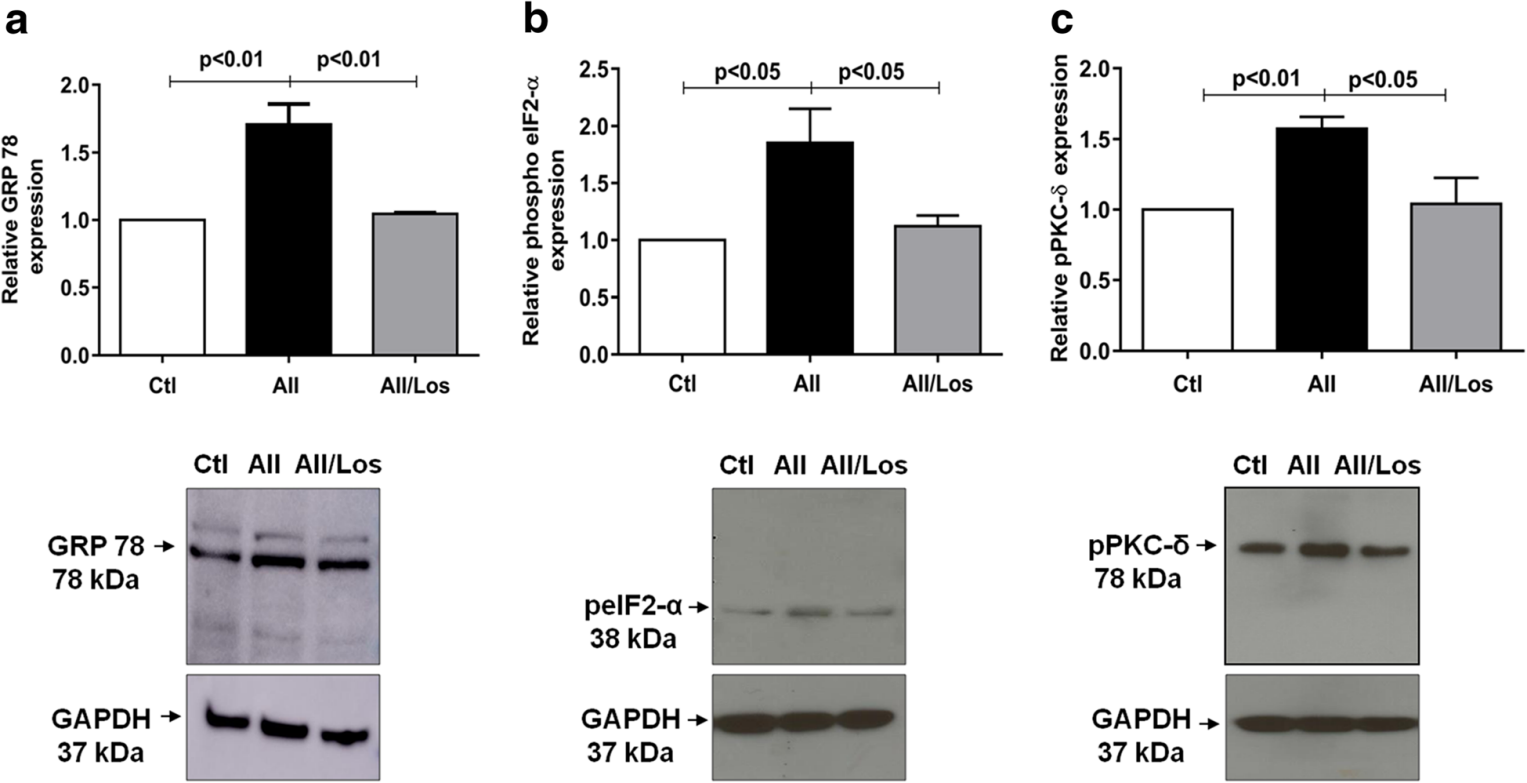
Relative expression and representative bands of GRP 78 (**a**), phosphorylated eIF2-α (peIF2-α) (**b**) and phosphorylated pPKC-δ (**c**) in control and treated podocytes. GAPDH was used as an internal control; values represent the mean ± SEM (*n* = 4–5 experiments)

Since Ang II induced an increase in glomerular and podocytes GRP 78 expression in vivo, we decided to further study its effects and the activated intracellular signaling pathways using an in vitro model of cultured podocytes. First, we confirmed that the differentiated mouse podocytes express podocin and AT1R (Fig. 2a, b). Next, we examined the effects of Ang II through AT1R on ER stress and apoptosis signaling pathways. 24 h Ang II treatment (1 μM) induced a significant increase in GRP 78 expression, enhanced the expression of an important ER stress component, eIF2-α, and also increased the phosphorylation of PKC-δ. All these Ang II effects were abrogated by losartan co-treatment (Fig. 3 and Table 1).

**Table 1:** Relative proteins expression and apoptosis in control and treated podocytes

	Ctl	AII (1 μM)	AII/Los (1 μM)	AII/SB (0.1 μM)	n
Protein					
GRP 78	1.00 ± 0.00	1.71 ± 0. 15^a^	1.05 ± 0.01^d^	–	4
peIF2-α	1.00 ± 0.00	1.87 ± 0. 21^b^	1.12 ± 0.09^f^	–	4
pPKC-δ	1.00 ± 0.00	1.59 ± 0.04^a^	1.05 ± 0.10^d^	–	5
pP38MAPK	1.00 ± 0.00	1.70 ± 0.03^a^	0.94 ± 0.06^d^	–	4
Total Bax	1.00 ± 0.00	1.64 ± 0.11^a^	–	0.95 ± 0.03^d^	4
Total Bcl-2	1.00 ± 0.00	1.19 ± 0.07	–	1.04 ± 0.03	4
Bax/Bcl-2 ratio	1.00 ± 0.00	1.39 ± 0.08^a^	–	0.90 ± 0.01^d^	4
Cell Viability %					
Viable	90.93 ± 0.70	87.34 ± 0.59^c^	–	92.65 ± 0.73^d^	5–6
Early apoptosis	0.62 ± 0.06	0.80 ± 0.07	–	0.43 ± 0.03^f^	5–6
Late Apoptosis	4.99 ± 0.28	6.99 ± 0.50^b^	–	4.61 ± 0.30^e^	5–6
Necrosis	3.43 ± 0.47	3.97 ± 0.36	–	2.48 ± 0.41	5–6

Values are means ± SEM. n, number of experiments. ^a^*p* < 0.001, ^b^*p* < 0.01 and ^c^*p* < 0.01 versus control (Ctl); ^d^*p* < 0.001, ^e^*p* < 0.01 and ^f^*p* < 0.05 versus angiotensin II (Ang II); *Los* losartan, *SB* SB203580; p phosphorylated protein expression

### Ang II stimulates p38 MAPK protein expression and apoptosis in podocytes

**Fig. 4:**
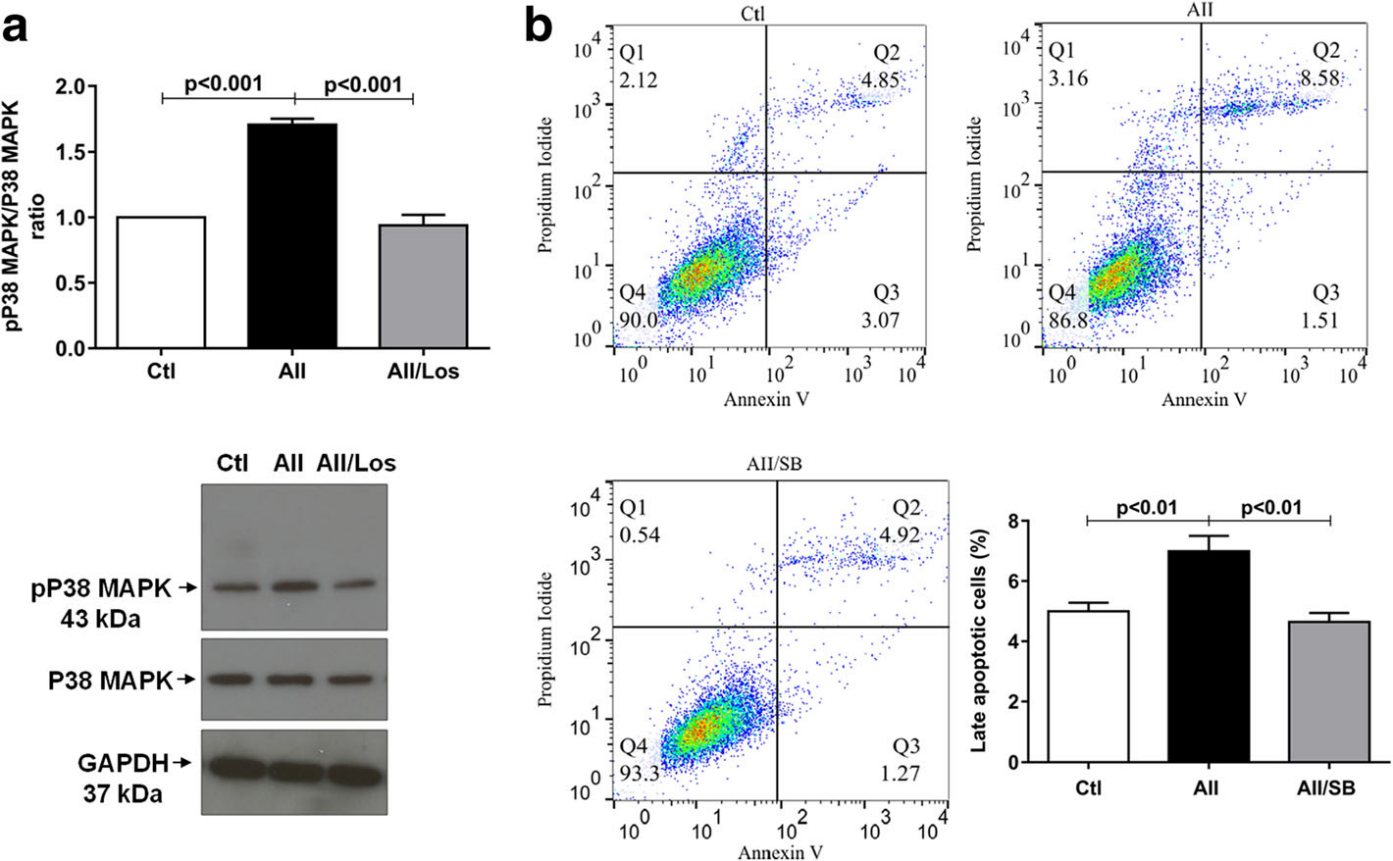
**a** Relative expression and representative bands of phosphorylated and non- phosphorylated p38 MAPK in control and treated podocytes. GAPDH was used as internal control; the values are mean ± S.E. of 4 experiments. **b** Podocyte apoptosis in control, Ang II (1 μM) and/or SB203580 (0.1 μM) treated cells, for 24 h, detected by flow cytometry using Annexin V/propidium iodide staining Q1, cells in necrosis; Q2, cells in late apoptosis; Q3, cells in early apoptosis and Q4, healthy cells. Late apoptosis quantification is expressed as mean ± SEM of 5–6 experiments, in triplicate

Because Ang II triggers ER stress and may have an important role in apoptosis, we evaluated the phosphorylation status of p38 MAPK and its contribution to apoptosis in Ang II-treated podocytes. As shown in Fig. 4a and Table 1, compared with control cells, podocytes treated with Ang II (1 μM) for 24 h showed significant increases in p38 MAPK phosphorylation, which was prevented by the AT1R antagonism. We next explored whether Ang II or p38 MAPK played a role in podocyte apoptosis. We observed that compared with control, chronic treatment with Ang II (1 μM) induced significant p38 MAPK-mediated decrease of podocyte viability, a slight increase of early podocyte apoptosis (29%) and significant late podocyte apoptosis. Podocyte necrosis was similar between both groups. These effects were prevented by SB203580, a specific p38 MAPK inhibitor (Fig. 4b and Table 1).

**Fig. 5:**
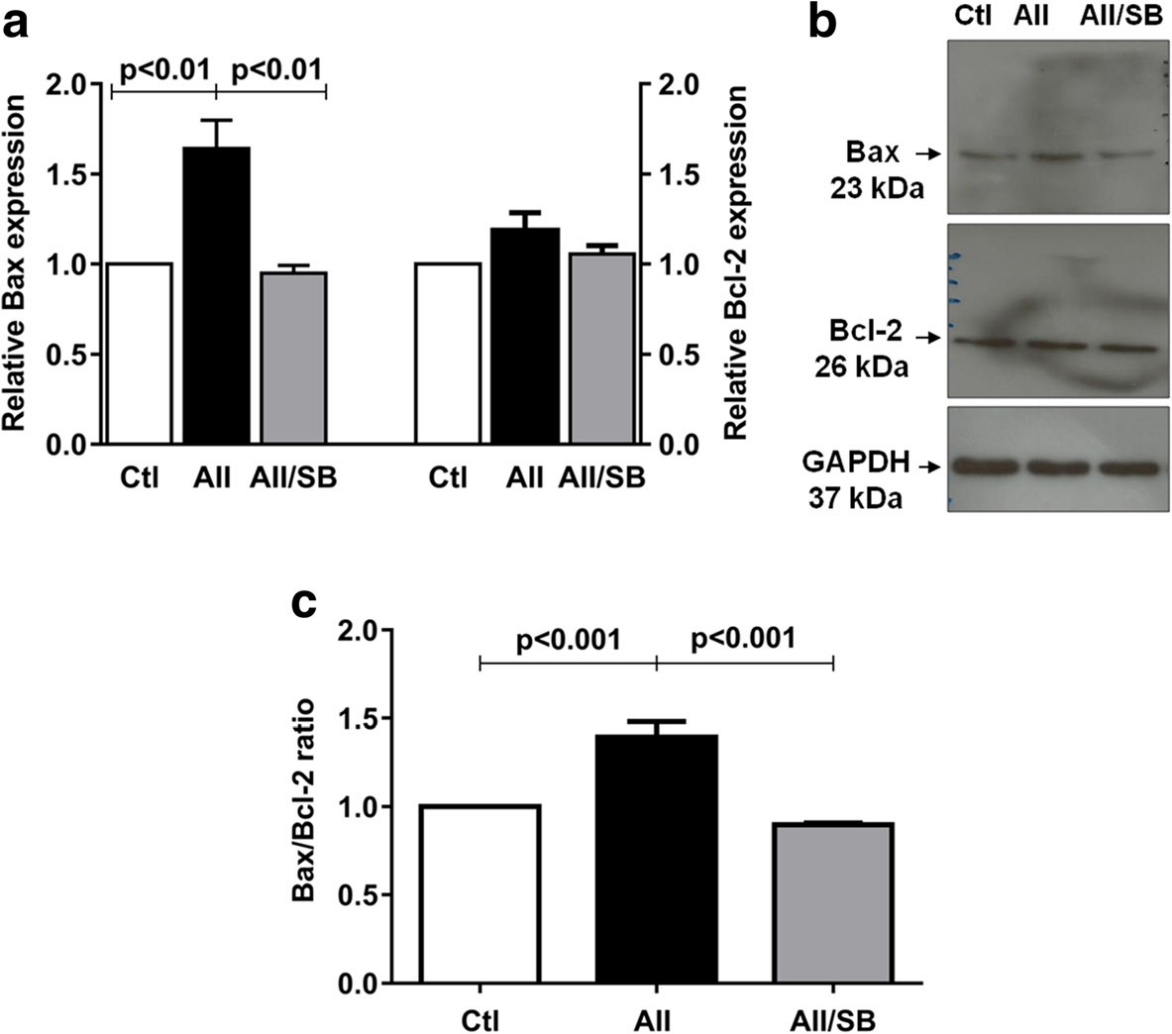
**a** Relative expression and **b** representative bands of Bax and Bcl-2 in control and treated podocytes. GAPDH was used as internal control. **c** Bax/Bcl-2 ratio. Values are mean ± SEM of 4 experiments

In addition, the protein expression analysis confirmed that phospho p38 MAPK-mediated the stimulatory effects of Ang II on Bax levels, which exceeded Bcl-2 when compared with controls. Consequently, the Bax/Bcl-2 ratio increased when compared with the control group (Fig. 5 and Table 1).

### Ang II-mediated p38 MAPK activation induces intracellular pH changes in podocytes

**Fig. 6:**
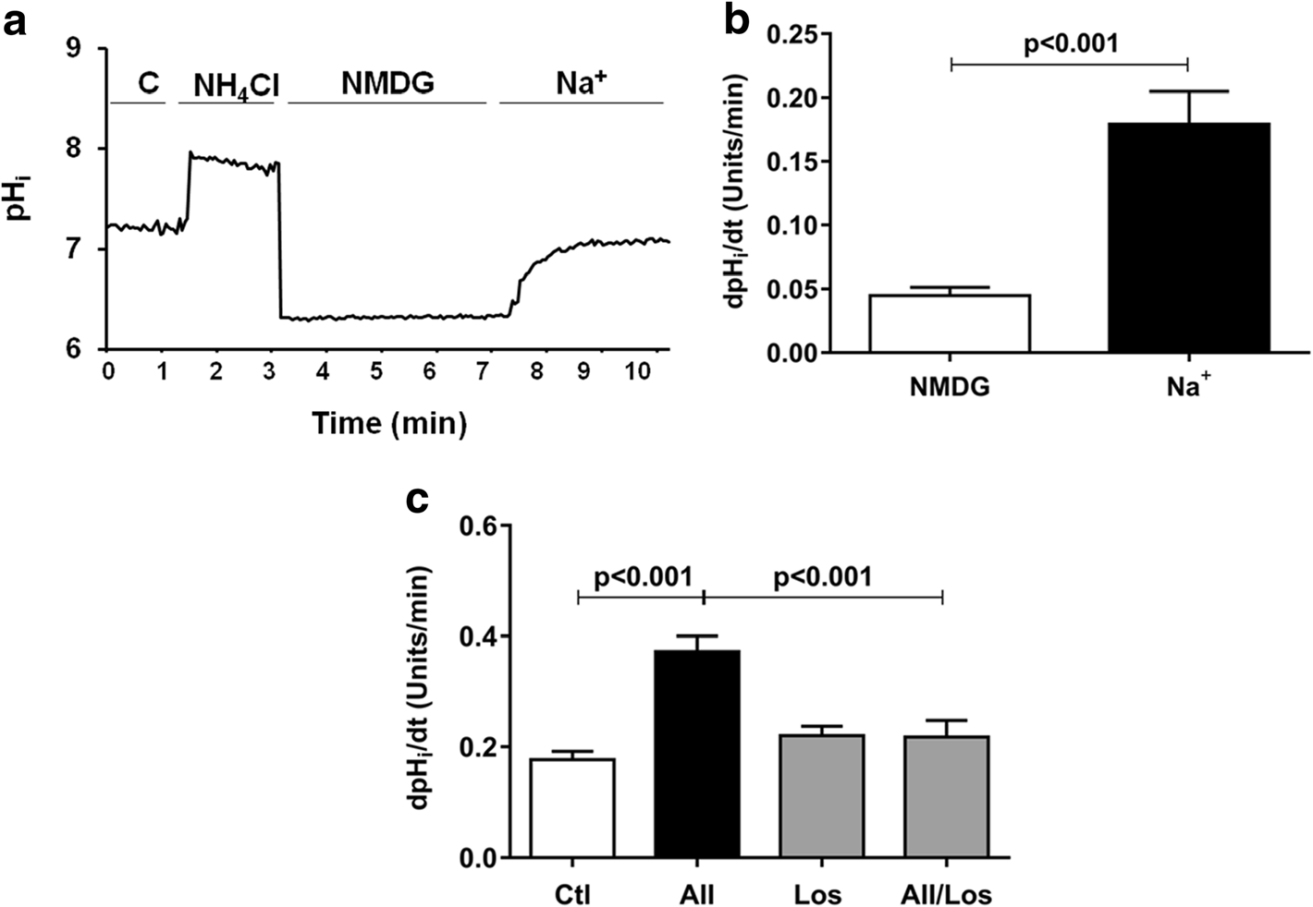
pHi recovery after acid loading. **a** Representative experiment of podocytes exposed to Na^+^-control solution, Na^+^-free solution with N-methyl-D-glucamine-NMDG (138 mM), followed by replacement of Na^+^-control solution (Na^+^ 138 mM). **b** pHi recovery rate using NMDG and Na^+^ solutions (138 mM). **c** The effects of Ang II (1 μM) and/or Losartan (1 μM) for 24 h on pHi recovery rate after acid loading in control and treated podocytes. The values are mean ± SEM of 8–11 / group

In renal tubular cells, acute treatment with Ang II has been shown to result in intracellular alkalinization [33]. Additionally, it is known that alkalinization may favor apoptotic events in other cells [23, 26, 27]. To distinguish among several membrane ion exchangers that could potentially induce intracellular alkalinization, we first evaluated sodium-dependent intracellular pH (pHi) recovery after acid loading, using the pH-sensitive probe BCECF. As shown in Fig. 6a, a representative trace of pHi recovery demonstrated that after acid loading with NH_4_Cl and 138 mM NMDG, the re-addition of a sodium solution induced pHi recovery to values approaching baseline levels. The mean of 11 experiments revealed that under control conditions, podocytes have a mean pHi baseline of 7.18 ± 0.01. This value increased to 7.69 ± 0.03 in the presence of NH_4_Cl, decreased to 6.5 ± 0.04 during acid loading and recovered to 7.11 ± 0.02 after the addition of Na^+^ solution.

Using linear regression analysis, the pHi recovery rate was calculated during recovery in the first 2 minutes in the absence (NMDG) or presence of sodium (Fig. 6b and Table 2). The results indicated that after acid loading, the mean pHi recovery rate in the solution containing NMDG was 0.045 ± 0.006 (pHi units/min). This value significantly increased after the addition of sodium solution, indicating that in podocytes, sodium is required for pHi recovery rate after acid loading. Then, we investigated whether extracellular Ang II (1 μM) acting via AT1R could affect the pHi recovery rate. As shown in Fig. 6c and Table 2, compared with control, podocytes treated with Ang II for 24 h showed significant increase in pHi recovery rate, which was normalized in Ang II/losartan cotreated cells. Losartan 1 μM alone did not change this parameter.

**Table 2:** pHi recovery rate in control and treated podocytes

	dpHi/dt (Units/min)		n
NMDG, 138 mM	0.045 ± 0.006		11
Control solution, Na^+^ 138 mM, added after NMDG solution	0.179 ± 0.025^a^		11
Control solution, Na^+^ 138 mM	0.176 ± 0.016		10
AII 1 μM	0.372 ± 0.028^c^		10
Losartan (1 μM)	0.220 ± 0.017		11
AII/Losartan (1 μM)	0.217 ± 0.029^d^		8
Cariporide (10 μM)	0.061 ± 0.006^b^		6
AII/Cariporide (10 μM)	0.067 ± 0.005 ^db^		10
SB203580 (0.1 μM)	0.177 ± 0.023		6
AII/ SB203580 (0.1 μM)	0.217 ± 0.036^e^		7

Values are mean ± SEM. n, number of experiments. ^a^*p* < 0.001 versus NMDG (N-methyl-D-glucamine); ^b^*p* < 0.001 versus control; ^c^*p* < 0.001 versus control; ^d^*p* < 0.001 versus angiotensin II (AII) and ^e^ *p* < 0.01 versus AII

**Fig. 7:**
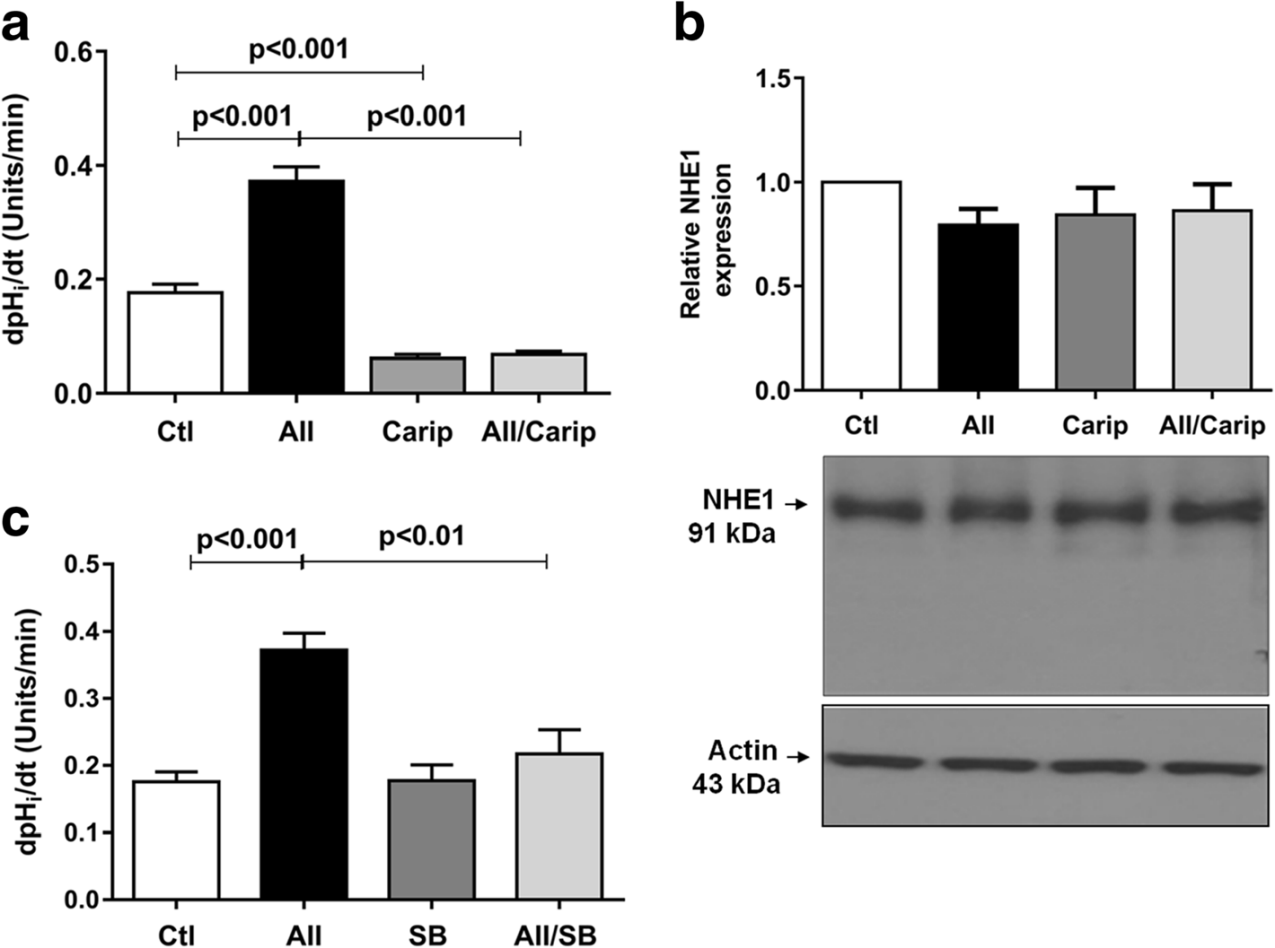
The effects of Ang II (1 μM) and/or cariporide (10 μM) (**a**) or SB203580 (0.1 μM) (**c**) for 24 h on pHi recovery rate after acid loading. NHE1 protein expression in control and treated podocytes (**b**). Values are mean ± SEM of 6–10 experiments/group

We also investigated whether the effect of Ang II/AT1R on pHi recovery rate was mediated through NHE1, by treating the cells with 10 μM cariporide, a specific NHE1 inhibitor for 24 h. Under this condition, the Na^+^-mediated pHi recovery rate significantly decreased when compared to both control and Ang II-treated groups (Fig. 7a and Table 2). However, the NHE1 protein expression remained unchanged (Fig. 7b) [(Relative NHE1 protein expression) Ctl, 1.00 ± 0.00; AII, 0.79 ± 0.07; Carip, 0.84 ± 0.13; AII/Carip, 0.86 ± 0.12 (*n* = 4)].

To examine the relationship between Ang II, p38 MAPK activation and intracellular alkalinization, we used a p38 MAPK inhibitor, SB203580 (0.1 μM). Compared with control, treatment with SB203580 alone did not change the pHi recovery rate. However, SB203580 prevented the increase in pHi recovery rate induced by Ang II (Fig. 7c and Table 2).

## Discussion

Podocytes play an essential role in maintaining the integrity of the GBM. Thus, podocyte injury is closely related to glomerular damage and proteinuria. RAS activation and the consequent high Ang II levels are important mediators of tissue damage in many pathological conditions, including hypertension and chronic kidney disease [6]. So, it is of sum importance to understand intracellular events associated with podocyte injury and apoptosis. To investigate the effect of Ang II on podocyte damage, we studied early events (e.g., ER stress) and signaling pathways (GRP 78, PKC-δ and p38 MAPK activation) that lead to NHE1-dependent cytosol pH changes and its relation to apoptosis.

Under physiological conditions, ER is a multifunctional organelle essential for the balance between ER protein synthesis and chaperone GRP 78-mediated protein folding [34]. GRP 78 binds to the luminal domain of ER sensor proteins such as inositol-requiring kinase alpha (IRE1-α), activating transcription factor 6 (ATF6) and double-stranded RNA-activated protein kinase (PKR)-like endoplasmic reticulum kinase (PERK) [35] and subsequently maintains their inactivated states. However, pathophysiological stimulus can disrupt ER homeostasis, resulting in the accumulation of misfolded and unfolded proteins and subsequently cellular toxicity [36]. This condition is known as ER stress and activates the unfolded protein response (UPR) pathway, which in association with GRP 78 can restore ER homeostasis [37]. However, over prolonged stimulus, GRP 78 preferentially binds to unfolded or misfolded proteins and dissociates from sensor proteins, favoring their activation [38, 39] and consequently regulates ER stress response genes [40]. Activated PERK initiates the phosphorylation of eukaryotic initiation factor 2 alpha (eIF2-α). In turn, phosphorylated eIF2-α activates transcription factor 4 (ATF4), which upregulates the expression of transcription factor CCAAT-enhancer-binding protein homologous protein (CHOP) and caspases, leading to apoptosis and tissue injury [41]. It has been reported that the Ang II plays an important role in the ER stress-induced renal apoptosis, including tubular cells and podocytes, especially in diabetic nephropathy [42, 43]. Ha et al. [44] showed that Ang II could induce podocyte ER stress via PERK-eIf2-α-ATF4 axis and PI3-kinase pathway. On this backgroud, using in vivo and in vitro approaches, we further studied the molecular signaling elicited by Ang II that culminates in podocyte apoptosis. Our results showed that, rats chronically treated with Ang II exhibited an AT1R-mediated increase of glomerular GRP 78.

Corroborating the in vivo finding, cultured podocytes treated with Ang II also showed increased GRP 78 expression, as well as increased eIF2-α phosphorylation, which were all abrogated with losartan co-treatment. These data provide new information that supports the association of Ang II/AT1R signaling and ER stress on podocyte injury.

Previous studies have demonstrated that protein kinase C delta (PKC-δ) also plays an important role in apoptosis. PKC-δ interacts constitutively with Abl, a non-receptor tyrosine kinase localized in the nucleus, cytoplasm and ER [45, 46]. However, under ER stress condition, the PKCδ-Abl complex translocates from the ER to the mitochondria to induce ER-stress-mediated apoptosis [47]. Finally, PKC-δ has been shown to interact with several members of the mitogen-activated protein kinase (MAPK) family, including p38 MAPK [48]. Indeed, in the present study, we observed that in podocytes, chronic treatment with Ang II induced significant increases in phosphorylated PKC-δ and p38 MAPK expression by an AT1R-dependent mechanism.

Regarding potential relationships between ER stress and p38 MAPK signaling in podocytes, it is known that in ER stress condition, p38 MAPK phosphorylates and activates the transcription factor CHOP (at Ser78 and Ser81), which favors apoptosis in the same cell lines [49–51]. Furthermore, p38 MAPK is reported to promote the phosphorylation and activation of pro-apoptotic protein Bax [52]. Our results with cultured podocytes were in agreement with these findings, since chronic treatment with Ang II induced an AT1R-dependent increase in the expression of the phosphorylated subunit of p38 MAPK. In addition, we observed that Ang II treatment induced p38 MAPK-dependent apoptosis in podocytes, which is associated with Bax protein activation.

Considering that p38 MAPK directly phosphorylates NHE1 [22, 23, 53], we speculated whether Ang II/p38 MAPK-dependent apoptosis signaling is also related to NHE1- mediated pHi changes in podocytes. NHE1 and other NHE isoforms promote cellular proton extrusion, using energy from a sodium gradient to catalyze the electroneutral exchange of 1 Na^+^ for 1 H^+^. Under physiological conditions, NHE1 is quickly activated by acid loading or osmotic cell shrinkage to maintain intracellular pH and volume homeostasis, ensuring cell survival. Moreover, NHE1 can also regulate other cellular events, including proliferation, migration and apoptosis [54, 55]; however, these mechanisms remain unclear. Whereas under apoptosis conditions, p38 MAPK can phosphorylate NHE1 at serine sites (Ser 726 and Ser 729) [23], we investigated the relationship between p38 MAPK and NHE1 in the apoptotic response induced by Ang II. In the current study, we observed a pHi baseline in podocytes of 7.18 ± 0.01 units, which is in agreement with other cell lines [26, 31]. We initially used NMDG to confirm the Na^+^-dependent pHi recovery rate after acid loading in podocytes. In addition, we used cariporide, a specific NHE1 inhibitor to confirm the NHE1 activity, and we also investigated whether NHE-1 is chronically activated by Ang II/AT1R signaling in podocytes. Our results revealed a significant stimulatory effect of Ang II via AT1R on pHi recovery rate after acid loading. Furthermore, our results demonstrated that NHE1 activity, rather than changes in its protein content, is essential for podocyte intracellular pH recovery after acid loading, since cariporide treatment significantly reduced the pHi recovery rate. Interestingly, the stimulatory effect of Ang II on pHi recovery rate after acid loading was prevented by SB203580. Therefore, our data suggest that Ang II/AT1R activate p38 MAPK pathway leading to NHE1 activation and consequently enhancing the pHi recovery rate after acid loading.

## Conclusion

**Fig. 8:**
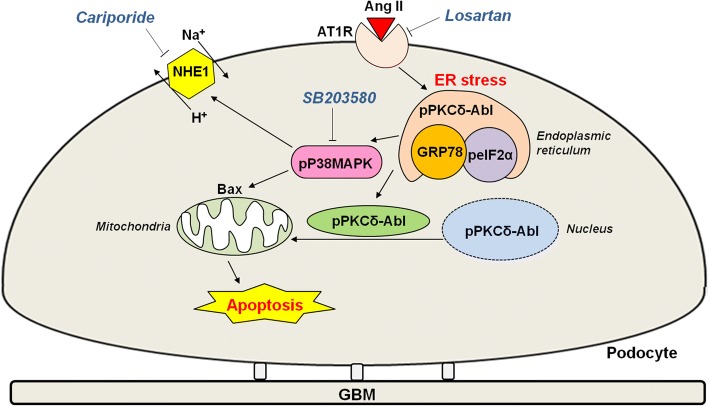
Mechanism by which Ang II induces apoptosis in podocytes. AT1R signaling induces ER stress through increased GRP 78 and p-elf2α expression and PKC-δ phosphorylation. p38 MAPK and PKC-δ activation lead to increased Bax expression and enhanced NHE1 activity, triggering cellular apoptosis. Cariporide, NHE1 inhibitor; Losartan, AT1R antagonist; SB203580, p38 MAPK inhibitor; GBM, glomerular basement membrane

In conclusion, our study provides new molecular mechanisms to Ang II-induced podocyte apoptosis, showing that chronic AT1R signaling induces ER stress/PKC-δ/p38 MAPK pathway activation. Activated p38 MAPK simultaneously stimulates pro-apoptotic Bax protein and NHE1 activity, favoring cellular apoptosis (Fig. 8).

## Abbreviations

Ang II: Angiotensin II
AT1R: Ang II AT1 receptor
BCECF/AM: (2′, 7′-bis-(2-carboxyethyl)-5-(and-6)-carboxyfluorescein, acetoxymethyl ester
eIf2-α: Eukaryotic initiation factor 2
ER: Endoplasmic reticulum
FITC: Fluorescein isothiocyanate
GBM: Glomerular basement membrane
IFN-γ: Interferon-gamma
NADPH: Nicotinamide adenine dinucleotide phosphate
NHE1: Na^+^/H^+^ exchanger isoform 1
p38 MAPK: p38 mitogen-activated protein kinase
pHi: Intracellular pH
PKC-δ: Protein kinase C delta
RAS: Renin-angiotensin system
ROS: Reactive oxygen species
SB203580: Pyrimidyl imidazole
Ser: Serine
